# Prenatal identification of an inverted duplicated 13q marker chromosome with a neocentromere

**DOI:** 10.1186/s13039-023-00666-w

**Published:** 2023-11-30

**Authors:** Liselot van der Laan, Daniel R. Hoekman, Esther J. Wortelboer, Marcel M. A. M. Mannens, Angelique J. A. Kooper

**Affiliations:** Department of Human Genetics, Amsterdam Reproduction and Development Research Institute, Amsterdam University Medical Centers, University of Amsterdam, Amsterdam, The Netherlands

**Keywords:** Neocentromere, Marker chromosome, False-negative NIPT, Placental mosaicism

## Abstract

In this case report, we describe a rare prenatal finding of a small marker chromosome. This marker chromosome corresponds to an inverted duplication of the 13q region 13q31.1q34 (or 13q31.1 → qter) with a neocentromere, detected during genetic analysis of a chorionic villus sample in a fetus with multiple congenital anomalies after a normal prenatal screening result by noninvasive prenatal testing.

## Background

The centromere is a chromosomal structure that allows cells to divide their genetic material during meiotic and mitotic cell divisions. Centromeres are usually characterized by tandemly repeated alpha satellite DNA and heterochromatin [1]. A neocentromere is a new centromere at a location, normally not centromeric, and are centromeric regions without alpha satellite DNA. They are able to form a primary constriction and assemble a functional kinetochore and carry out the function of a normal centromere. This shows that centromere formation is not only dependent on primary DNA sequence [2]. The majority of neocentromeres in marker chromosomes are an inverted duplication of the distal parts of a chromosome arm resulting in partial tri- or tetrasomy [3]. A small supernumerary marker chromosome (sSMC) is an additional chromosome that usually lacks a distinct banding pattern and which can be rarely identified by conventional banding [4]. Therefore additional techniques are necessary to characterize the origin of the marker chromosome. We present a case of a sSMC originating from an inverted duplication of distal 13q31.1q34 found in a chorionic villus sample (CVS) in a fetus with multiple congenital anomalies. Previously, the 31 year-old pregnant woman received a normal screening result of noninvasive prenatal testing (NIPT). Both cytogenetic and molecular techniques were used to characterize this finding.

## Case presentation

**Fig. 1:**
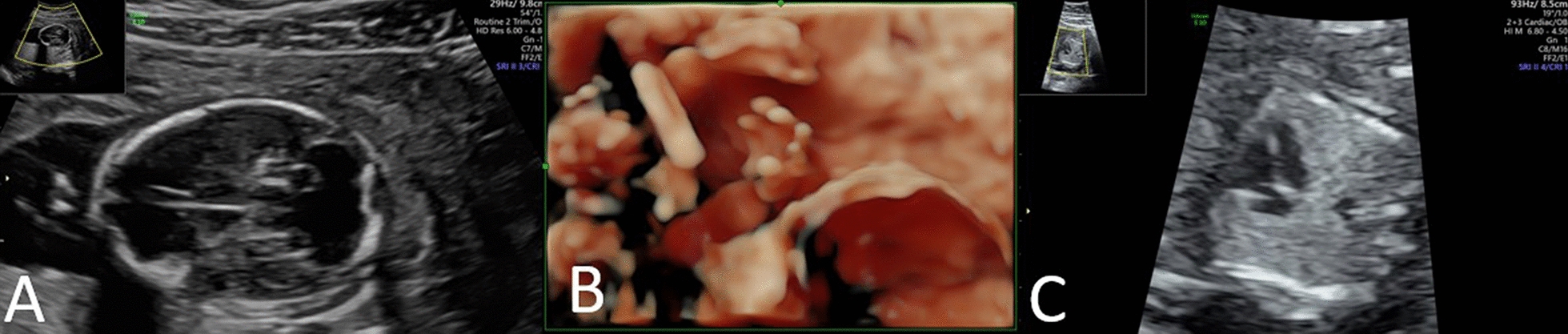
Ultrasound anomaly scan **A** Midline defect cerebral (compatible with holoprosencephaly) **B** Polydactyly and **C** Congenital cardiac defect (suspect for ventricular septal defect)

We present a case of a primigravida (conceived by intracytoplasmic sperm injection) who underwent targeted NIPT in the Dutch TRIDENT-2 study [5] with a normal result for chromosomes 13, 18 and 21. At 14 + 3 weeks of gestation, a routine structural ultrasound scan revealed multiple anomalies: a cerebral midline defect (compatible with holoprosencephaly), polydactyly, and a congenital cardiac defect (Fig. 1). Chorionic villus sampling was performed. Based on the ultrasound anomalies, the pregnancy was terminated at 17 weeks of gestation. Postpartum physical examination of the fetus showed leg contractures and polydactyly of the feet. Fetal autopsy was not performed.

## Material and methods

Previously, NIPT was performed as part of the Dutch TRIDENT-2 study (Trident = Trial by Dutch laboratories for Evaluation of NIPT). The method that was used shortly involved genome‐wide shallow massively parallel shotgun sequencing and genome‐wide analysis with WISECONDOR that has a resolution of approximately 15 Mb at a sequencing depth of about 10 to 12 million reads per sample.

Sex chromosomes were not analyzed. Parents choose to have a targeted NIPT analysis for the chromosomes 13, 18 and 21.

After receiving the CVS, Trypsin Express was used to remove the cytotrophoblasts layer. Cytotrophoblast cells were stored. Subsequently, routine Quantitative Fluorescence Polymerase Chain Reaction (QF-PCR) and array-CGH analyses were conducted on DNA isolated from the mesenchymal core cells of the villi. In parallel, cell culture on the mesenchymal core cells was performed in duplicate using two different cell culture media (Amniomax C100 and Chang D medium), according to standard procedures of the Amsterdam UMC, Human Genetics department. QF-PCR analysis on DNA from the mesenchymal core cells was performed using the QST*Rplusv2 kit from Elucigene (Manchester, UK) following the instructions of the manufacturer. Interpretation of the data was performed according standard and accredited procedures of the Amsterdam UMC, Human genetics department.

Array-CGH microarray analysis was performed on DNA from the mesenchymal core cells using 180 k microarray slides from Agilent Technologies (Santa Clara, CA), following manufacturer’s protocols using a mixed pool of 50 healthy males as a reference. Scanned images were analyzed by using Agilent Genomic Workbench version 6.5 and Cartagenia (Agilent Technologies). Interpretation of CNV data was performed as described in the guidelines of the lab.

Fluorescence In Situ Hybridization (FISH) was performed according to standard procedures of the Amsterdam UMC, Human Genetics department. The following probes were used; centromere 12 (CEP12), LSI D13S319 (13q14.3) and LSI 13q34 from Vysis Abbott (Inc, London, United Kingdom).

## Results

**Fig. 2:**
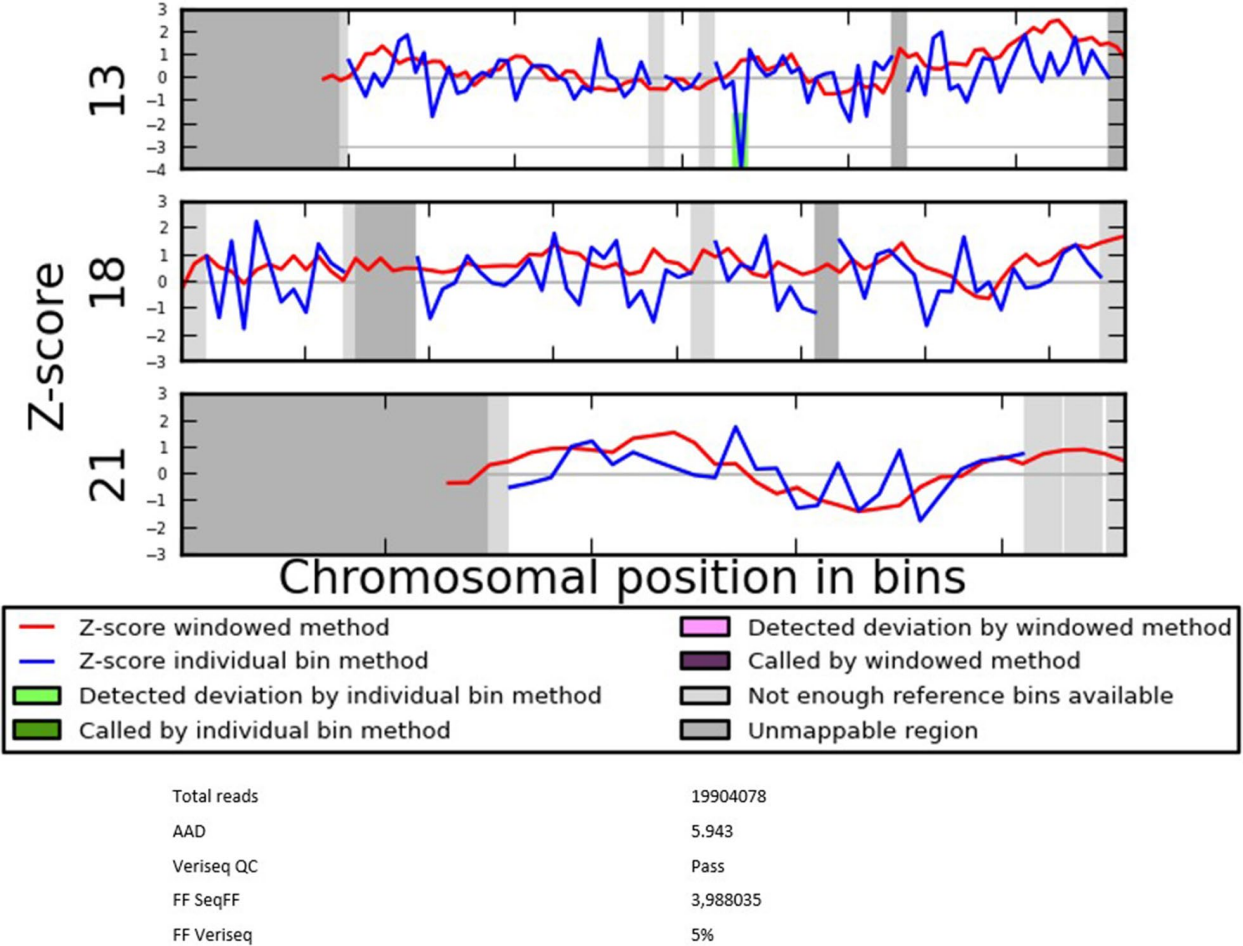
WISECONDOR plot showing the normal NIPT result for the chromosomes 13, 18 and 21

Parents received a normal result of the NIPT screening. The corresponding NIPT profile is shown in Fig. 2.

**Fig. 3:**
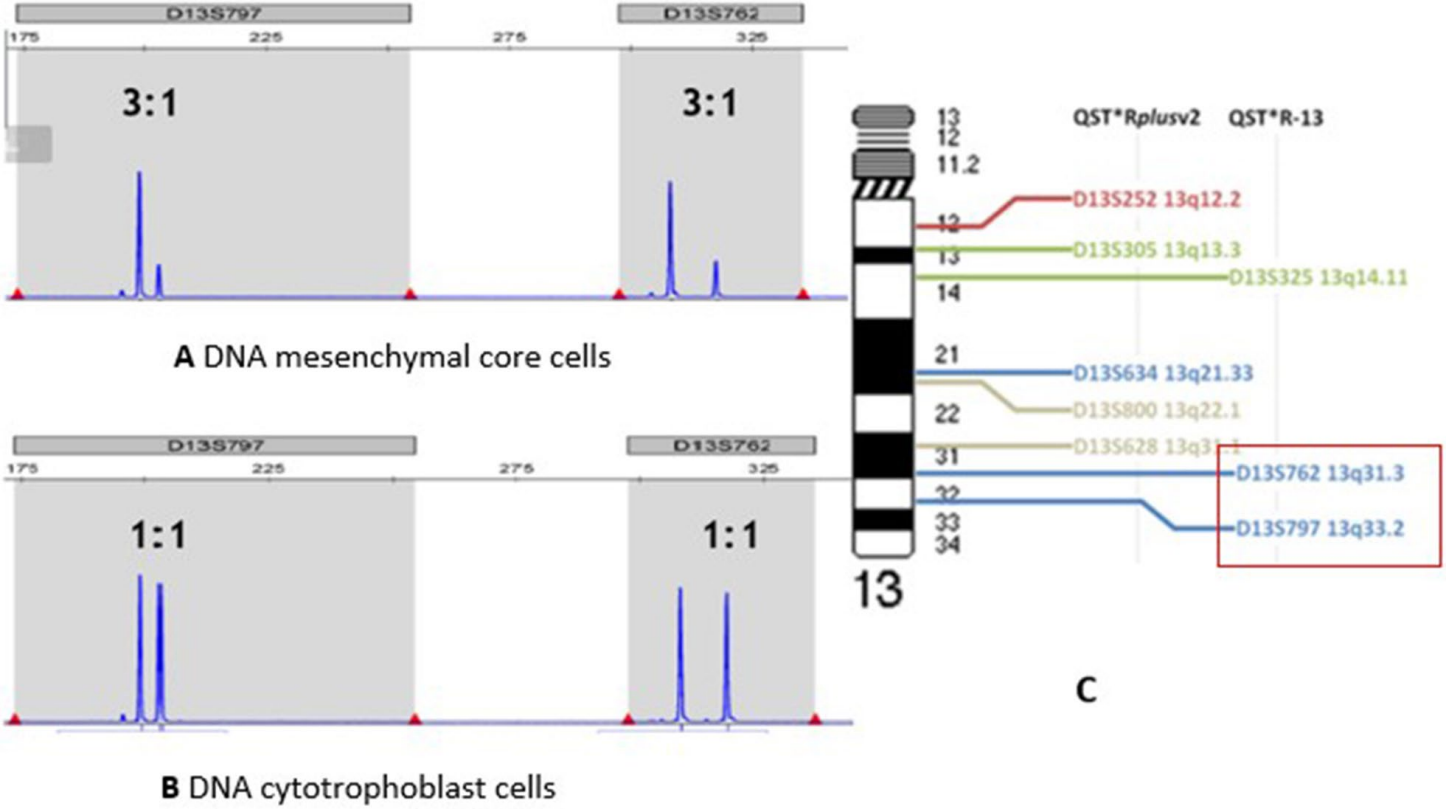
**A** 3:1 ratio for the STR markers D13S797 and D13S762 in DNA from mesenchymal core cells. **B** 1:1 ratio for D13S797 and D13S762 in DNA from cytotrophoblasts cells. **C** STR marker location, idiogram STR markers for chromosome 13. Only the framed markers are abnormal

The QF-PCR profile on DNA from the mesenchymal core cells was normal for chromosomes 18, 21, X and Y consistent with a male profile. However, due to insufficient informative markers for chromosome 13, an additional kit for chromosome 13 (QST*R- 13) was used. Unexpectedly, two QF-PCR markers D13S762 and D13S797, located in the 13q terminal region, showed an abnormal profile with a 3:1 ratio (Fig. 3A). In conclusion, there are two abnormal markers in the terminal region, two normal markers for chromosome 13 (D13S800 and D13S325), whereas the other four markers for chromosome 13 were not informative.

**Fig. 4:**
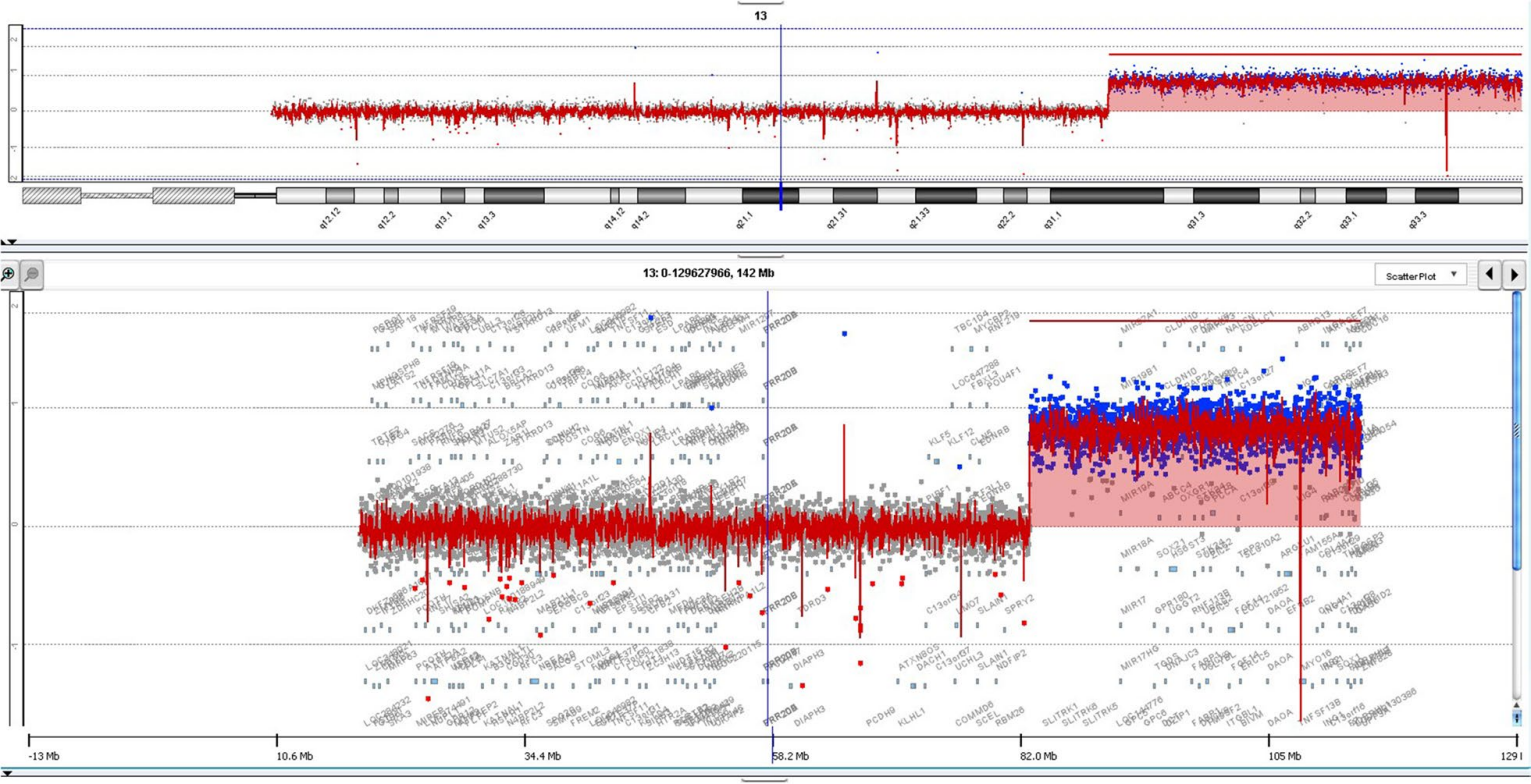
Array-CGH profile of chromosome 13 showing the homozygous copy number gain of approximately 31.7 Mb in the distal part of the long arm of chromosome 13: arr[GRCh37] 13q31.1q34(83416907_115105806) × 4 (hg19). The upper part of the figure displays chromosome 13, and below it, there is a zoomed-in view of chromosome 13

Subsequently, array-CGH was performed, revealing a homozygous copy number gain of approximately 31.7 Mb in the distal part of the long arm of chromosome 13 (13q31.1q34). This region contains multiple OMIM morbid genes (*SLITRK1, SLITRK6, GPC6, DCT, TGDS, CLDN10, DZIP1, DNAJC3, ZIC2, PCCA, NALCN, FGF14, TPP2, ERCC5, SLC10A2, DAOA, LIG4, IRS2, COL4A1 COL4A2, NAXD, CARS2, ING1, ATP11A, F7, F10, PROZ, GRK1, CHAMP1*) (Fig. 4). This finding corresponded with the earlier result of the QF-PCR test.

**Fig. 5:**
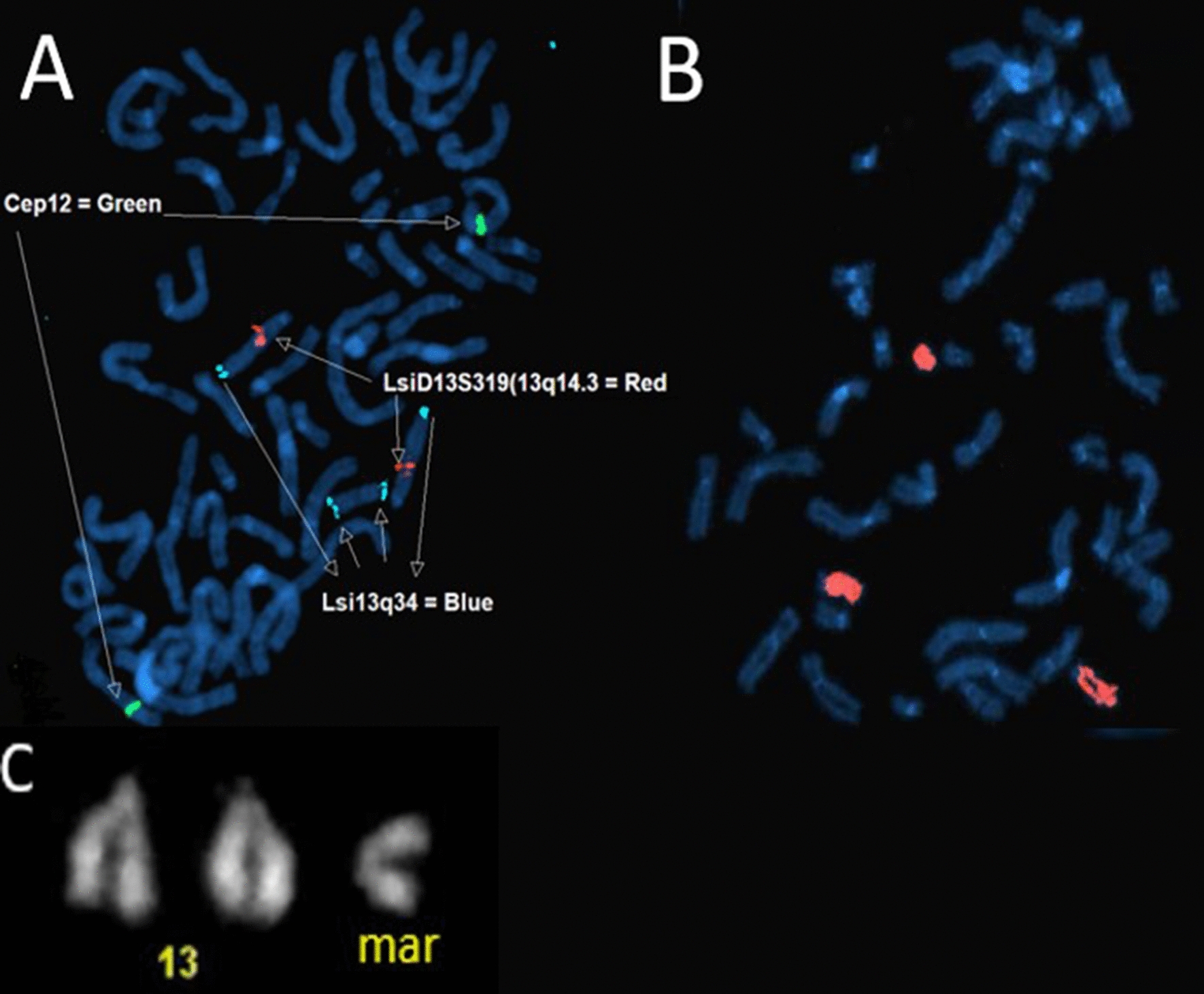
The FISH results **A** Metaphase FISH analysis was performed using the LSI D13S319 (13q14.3, red) and LSI 13q34 (blue) probes from Vysis Abbott and a centromere probe for chromosome 12 (CEP12) was used as a control probe. **B** The whole chromosome painting probe for chromosome 13 (WCP13) painted both normal chromosomes 13 and the marker chromosome, indicating the marker chromosome originates from chromosome 13. **C** The normal chromosomes 13 and the marker chromosome stained by quinacrine banding (Q-band)

Additional karyotyping of cultured mesenchymal core cells revealed a small sSMC in all metaphases with a centromere located in a non-centromere region (neocentromere). The normal chromosomes 13 and the marker chromosome stained by quinacrine banding (Q-band) are shown in Fig. 5C.

To confirm that the marker chromosome consisted of the duplicated region of chromosome 13 detected by array analysis, metaphase FISH analysis was performed using a whole-chromosome painting probe for chromosome 13 (WCP13) and a probe for the region 13q34 (13qter). A centromere probe for chromosome 12 (CEP12) was used as a control probe for euploidy. The WCP13 probe painted both normal chromosomes 13 and the marker chromosome, indicating the marker chromosome originates from chromosome 13. Subsequently, the probe for the region 13q34 (13qter) showed a signal at both distal regions of the marker chromosome, confirming the partial tetrasomy of 13q originated from an inverted duplicated neocentric marker chromosome derived from the distal region of 13q (Fig. 5). In total 50 metaphases were examined.

To address the discrepancy between the normal NIPT screening result and the abnormal array finding of the mesenchymal core cells in the CVS, DNA isolated from the cytotrophoblasts cell suspension was examined by QF-PCR with markers for chromosome 13. All informative markers for chromosome 13 showed a normal pattern (ratio 1:1) (Fig. 1b).

## Discussion

We report a CVS that showed a partial tetrasomy of distal 13q resulted from an inverted duplicated neocentric marker chromosome 13. This aberration was found with QF-PCR and array-CGH and later confirmed with karyotyping and FISH in the mesenchymal core cells of the CVS.

Previously, the couple underwent NIPT screening with a normal test result. During routine cytogenetic testing of CVS, in our laboratory the cytotrophoblasts layer is removed and stored as a backup, while diagnostic testing is conducted on the mesenchymal core cells. The partial tetrasomy of distal 13q appeared to be present in the mesenchymal core cells but not in the cytotrophoblasts. These two opposing cytogenetic constitutions of the fetoplacental compartments result in a false-negative NIPT screening result [6, 7]. Due to the severity of the ultrasound anomalies, the parents terminated the pregnancy without consent for autopsy or additional postpartum testing.

Partial tetrasomy 13q has been described prenatally in the literature only twice. Mascarenhas et al. and Hadad et al. both described a fetus with a sSMC consisting of an inverted duplication of the distal portion of chromosome 13q [8, 9]. The molecular and clinical findings in those two cases are compared with our case in Table 1.

**Table 1:** Molecular and clinical data in a fetus with partial tetrasomy 13q

	Mascarenhas et al. [8]	Haddad et al*.* [9]	Present case (2023)
Region	q31-qter	q31-qter	q31.1-qter
Neocentromere location	13q31	13q31	13q31.1
Mosaicism (%)	100	100	100
Sex	F	M	M
Gestational age	23 WG	17 WG	17 WG
Growth delay	NA	+	−
Microcephaly	NA	−	−
Macrocephaly	NA	NA	+
Hypertelorism	−	−	−
Hypotelorism	+	−	−
Nose abnormalities	+	+	−
Philtrum	NA	−	−
Cleft palate/lips	−	−	−
High palate	−	−	NA
Microphthalmia	−	−	−
Coloboma	NA	−	NA
Abnormal ears	+	+	−
Heart defects	NA	−	+
Lung defects	NA	−	−
Gastrointestinal malrotation	NA	+	−
Polydactyly	NA	+	+
Skeletal abnormalities	NA	+	+
Genital abnormalities	NA	+	−
Renal defect	+	+	NA
Cerebral midline defect	NA	NA	+

*NA* not available, *WG* weeks of gestation

We do not see an overlap with the cases from Mascarenhas et al*.* and Hadad et al*.* There was no autopsy performed in our case and therefore it might be possible that some anomalies remained undetected. Liehr et al*.* describes postnatal patients with sSMC of chromosome 13 but they are all present in mosaic and do not overlap in phenotype with our case [4]. In general partial tetrasomy of 13q has a reported association with a variable phenotype including microphthalmia, ear abnormalities, hypotelorism, facial dysmorfisms, urogenital defects, pigmentation and skin defects, and severe learning difficulties [10–13].

The formation of a neocentromeric marker of distal 13q, band 13q31 appears to be a known hotspot for neocentromere formation [14, 15]. In our case this additional sSMC was part of a mosaic placenta, present in the mesenchymal core cells, but absent in the trophoblasts. While we found no evidence of mosaicism in the mesenchymal cells of the villi, it is noteworthy that we did not assess the genotype of the fetus, as access to amniotic fluid or a postpartum skin biopsy was unavailable. Consequently, the potential existence of a mosaicism in the fetus cannot be ruled out and might explain the different phenotype.

This case also illustrates that a normal NIPT screening result does not exclude chromosomal aberrations in a fetus and shows the importance of standard ultrasound examination during pregnancy to improve the detection rate of fetal chromosomal abnormalities.

## Conclusions

We describe a fetus with a partial tetrasomy 13q resulting from an inverted duplicated neocentric marker chromosome. The marker chromosome was present in the mesenchymal core layer and absent in the cytotrophoblasts of the villi, and therefore remained undetected by NIPT.

## Data Availability

Data are available from the authors on request.

## Abbreviations

sSMC: Small supernumerary marker chromosome
CVS: Chorionic villus sample
NIPT: Non-invasive prenatal test
NA: Not available
WG: Weeks of gestation
WCP13: Whole-chromosome painting probe for chromosome 13
TRIDENT-2: Trial by Dutch laboratories for evaluation of NIPT
QF-PCR: Quantitative fluorescence polymerase chain reaction
